# Case of Alkohol Administered to a Man after Having Taken an Immoderate Dose of Opium

**Published:** 1808-06

**Authors:** 

**Affiliations:** New-York


### *Case of* ALKOHOL *administered to a Man after having taken an immoderate Dose of* OPIUM: *by Mr.* SHEPHARD, *of New-York.*

[ Extracted from the New-York Medical Repository. ]

I SUBMIT to your consideration a case of debility, arising from the effects of Opium, that fell under my care, which, perhaps, more accurately illustrates the operation and effects of *this* medicine, and shows to what extent *it* may be exhibited, and the patients still have a chance for recovery, than almost any experiment by which it has been preceded.

Daniel Grayham, aged about 35 years, of a firm, robust habit, and apparently in perfect health, residing at the time transiently in Schenectady, in this State, took the fatal resolution of destroying himself, and made the desperate attempt by the means of opium. Early on the morning of February 4, 1800, he procured, of a physician in that town, two drachms of opium; and then, repairing to an adjacent inn, called for a bed (complaining of illness), and immediately took the intended fatal portion. This, as nearly as could be ascertained, was about eight o'clock in the morning; and about eleven he was discovered by one of the family. I saw him immediately, and found him in a state of insensibility, accompanied with a complete apoplectic respiration, and some slight convulsive contractions of the muscles, especially of the hands and face. The pulse was full, regular, and not perceptibly removed from the standard of health. From these appearances I supposed him to be in the interval of an epileptic paroxysm; it not being known, at this time, that he had taken any thing to injure himself, or even had the design in contemplation. The symptoms, however, continuing the same for a considerable length of time, without any succession of spasm, or appearance of returning animation, I was totally at a loss how to proceed, or, in fact, what opinion to form of the disease. Some other physicians were now called in by the general alarm; but, previous to their arrival, I had accidentally discovered a paper upon the floor, in which, I found, had been opium; and which, at once, developed the wretched scene before me.

One of the physicians who now attended proposed, on this discovery, to immediately give an emetic, which was accordingly exhibited: but as the opium had had its full effect long before, no benefit could be expected from *such* practice. I exerted myself to have an opposite mode of practice pursued, and could not effect my purpose; but calmly contented myself with looking on, while a variety of other exertions were made, without the least advantage.

As a dernier resort, he was, about two o'clock P. M. placed into the hot-bath, in which he was immersed a considerable, and, probably, a sufficient length of time; but the result of this experiment was similar to every former trial; and he was now abandoned by all to the certainty of his fate.

For some time previous to this period I had been absent; but about four o'clock, and near two hours after all exertions in the patient's favour had been discontinued, I again saw him.

To support the system by the exhibition of an *adequate* degree of stimuli was the object I had before wished to pursue. In *this* last stage of the disease it was the *only* object. And although I scarcely retained a hope of success, yet the dictates of humanity, the prospect of future utility to the world, and the confidence I had in the principles on which I proceeded, urged me not to relinquish every hope while existence remained. *This* was the moment to which the the stimulant mode of cure was peculiarly *adapted*; for now the effects of the opium were subsiding, and the system rapidly sinking into the impending dissolution with which it was momentarily threatened. The apoplectic respiration now nearly suspended, and a weak, sinking, tremulous and irregular pulse, were the only indications of remaining vitality. His skin was universally cold, and partially suffused with a glutinous moisture; and his cadaverous countenance was literally the aspect of death. The tongue and mouth (which were widely extended) were perfectly dry, and produced, to the touch, a sensation similar to that arising from the action of a rasp; and, in fact, every appearance pointed to an immediate termination of the tragical scene. These were the formidable symptoms I had to encounter; and, as the patient was exposed to public view, I sustained a degree of censure for the temerity of retaining a hope of success in a situation so desperate. The safety of the patient, and my own reputation, were sources of great anxiety; but still I adhered to my first resolution of trying the experiment.

I took some brandy, which having sweetened, and diluted with a trifle of water, I exhibited a little immediately after each respiration, the intervals of which were now near a minute. I soon found the mouth and tongue were assuming a degree of natural softness, and was confident the liquid was gradually, though quite mechanically, passing into the stomach; and in this cautious manner I very soon gave more than a gill of the brandy; and I flattered myself I discovered some slight symptoms of returning animation. At this period I was obliged to leave the patient with an attendant, directing him, with the most forcible injunctions, to steadily pursue the plan I had adopted. I was absent near an hour; and, on my return, had the pleasing satisfaction of finding my patient perfectly sensible, able to turn and raise himself in bed, and to converse freely; but incapable of supporting an erect position, in consequence of the vertigo incident to that situation, in cases of extreme debility.

He had now taken about one pint of brandy. His irritability was so great, that the smallest cause of emotion would excite great, and sometimes an alarming degree of agitation. I continued this practice through the night, without any variation, excepting gradually diminishing the quantity of stimulus exhibited. The next morning I changed the stimulus by alternating the brandy with wine; which I continued through the day and night ensuing, still dimi-

 nishing nishing the quantity of spirits; and, as soon as the stomach would admit, gave him biscuit, and wine and water, and from thence proceeded to the use of fresh-meat broths. On Thursday he took a portion of calomel; which, I presume, from the irritable state of the stomach, proved violently emetic, and very slightly cathartic.

This medicine was materially serviceable, as his lungs had been all along somewhat oppressed; which, by this, was relieved, and the stomach and bowels sufficiently cleansed for the reception of the bark. From this period the bark was exhibited, and the laudanum and wine used as stimuli; and the case, in every respect, treated as a convalescent of indirect debility. In about ten days he was able to leave the town, which he performed on foot.

After his recovery he informed me, that his design being to make his exit sure, he had taken all the opium procured of the physician above-mentioned, which he said was *two drachms* (the truth of which the physician afterwards confirmed to me), together with a piece he had before obtained, as he supposed about half a drachm; and that he retained his recollection for about ten minutes after taking the dose.

One observation occurred to me, which, I presume, will be found of consequenc in the treatment of similar cases; which is, that *no* stimuli should be exhibited till the effects of the one constituting the disease are subsiding, and the system discovers evident marks of sinking. This is the period to employ the assistance of other stimuli, to give due support to the habit, and to excite and maintain *its* action till the morbid disturbance has *ceased to be.* Had the brandy been given on the first discovery of the patient, undoubtedly the already too highly excited action would have been increased, the hopes founded on this practice frustrated, and the patient lost. There is room sufficient for observations, and for a good deal of theoretical speculation, on a case so singular as the above: but, as I was under the necessity of protracting it to the length in which it now appears, I presume that further remarks may be dispensed with.

